# Acute gut inflammation reduces neural activity and spine maturity in hippocampus but not basolateral amygdala

**DOI:** 10.1038/s41598-022-24245-y

**Published:** 2022-11-23

**Authors:** Chelsea E. Matisz, Nadia Semenoff, Al-Shaimaa F. Ahmed, Lateece Griffin, Laurie E. Wallace, Parker McNabb, Robbin Gibb, Keith A. Sharkey, Aaron J. Gruber

**Affiliations:** 1 Canadian Center for Behavioural Neuroscience, Department of Neuroscience, University of Lethbridge, 4401 University Drive West, Lethbridge, AB T1K 3M4 Canada; 2 Department of Physiology & Pharmacology, Cumming School of Medicine, Hotchkiss Brain Institute and Snyder Institute for Chronic Diseases, University of Calgary, Calgary, AB Canada; 3 Department of Pharmacology & Toxicology, Faculty of Pharmacy, Minia University, Minia, Egypt

**Keywords:** Neuroscience, Physiology, Diseases, Gastroenterology

## Abstract

Gastrointestinal tract (gut) inflammation increases stress and threat-coping behaviors, which are associated with altered activity in fear-related neural circuits, such as the basolateral amygdala and hippocampus. It remains to be determined whether inflammation from the gut affects neural activity by altering dendritic spines. We hypothesized that acute inflammation alters dendritic spines in a brain region-specific manner. Here we show that acute gut inflammation (colitis) evoked by dextran sodium sulfate (DSS) did not affect the overall spine density in the CA1 region of hippocampus, but increased the relative proportion of immature spines to mature spines on basal dendrites of pyramidal neurons. In contrast, in animals with colitis, no changes in spine density or composition on dendrites of pyramidal cells was observed in the basolateral amygdala. Rather, we observed decreased spine density on dendrites of stellate neurons, but not the relative proportions of mature vs immature spines. We used cFos expression evoked by the forced swim task as a measure of neural activity during stress and found no effect of DSS on the density of cFos immunoreactive neurons in basolateral amygdala. In contrast, fewer CA1 neurons expressed cFos in mice with colitis, relative to controls. Furthermore, CA1 cFos expression negatively correlated with active stress-coping in the swim task and was negatively correlated with gut inflammation. These data reveal that the effects of acute gut inflammation on synaptic remodeling depend on brain region, neuronal phenotype, and dendrite location. In the hippocampus, a shift to immature spines and hypoactivity are more strongly related to colitis-evoked behavioral changes than is remodeling in basolateral amygdala.

## Introduction

Diseases with peripheral inflammation, such as inflammatory bowel disease (IBD), are strongly comorbid with mood disorders. For example, an estimated 20–32% of patients with IBD are diagnosed with anxiety disorders and 15–25% of patients suffer with depression^1,2^. Furthermore, anxiety and depression are associated with greater disease activity in IBD patients^3^. Animal models of gut inflammation are also associated with a variety of anxiety-like behaviours^4^. Such behaviours are influenced by contextual information and stimuli encoded by multiple neural circuits, particularly the hippocampus (HPC) and basolateral amygdala (BLA)^5^. Although it remains unknown how gut inflammation affects the neuropil in these structures, systemic peripheral inflammation evoked by injection of lipopolysaccharide leads to structure-specific changes in the density and morphology of dendrites and synapses in these structures^6–8^. Combined, these studies suggest that anxiety-like behaviours evoked by gut inflammation may arise from differential synaptic remodeling in HPC and BLA. Such region-specific changes have also been reported in models of chronic stress^9–12^, although the role of inflammation remains poorly understood in these models.

One of the most commonly used animal models of IBD is the addition of dextran sodium sulfate (DSS) in drinking water (2–4%) of mice^13^. This produces inflammation in the colon, and elevations of pro-inflammatory cytokines in the circulation that affect expression of neurotrophic factors and elevate pro-inflammatory cytokines in the brain^14–20^. These signaling molecules promote changes in the morphology of neurons, particularly the branching of dendrites and the density and shape of dendritic spines^21–23^. This may, in principle, partly account for the hyperactivity observed in threat-coping neural structures in models of gut inflammation^5^. Surprisingly little is known about how gut inflammation affects spine morphology; to our knowledge, no studies have yet characterized this in IBD models.

Dendritic spines are small protuberances from dendritic trunks that form compartments specialized for synaptic connections. They are highly plastic structures. Mature spines possess a ‘mushroom-like’ or stubby appearance and form strong synaptic connections. Occasionally, mature dendritic spines will also exhibit bifurcation^24^. Some evidence suggests spine bifurcation is correlated with long-term potentiation, possibly represents locus for synaptic modifications, and possesses altered efficacy of synaptic transmission^25^. Their immature counterparts lack a distinctive head and appear longer and thinner. Whereas mature spines nearly always form a functional synaptic connection, immature spines may or may not form a synapse with an innervating axon^26^. Immature spines, therefore, appear as placeholders for future connections, and can develop into mature spines as a consequence of learning via activity-dependent potentiation^27^. Both spine density and spine maturity (indicated by morphology) affect neuronal excitability^12,26^. Because the majority of spines in the neocortex are glutamatergic, and therefore excitatory, an increase in either spine density or relative fraction of mature spines will increase neural activity if other physiological properties of the cell (e.g., input resistance) are not much affected.

Changes in spine morphology have been studied more extensively in models of chronic stress than in inflammation. Interestingly, chronic restraint stress was associated with dendritic atrophy in the CA1 of the HPC, and increased dendritic arborization in the BLA; conversely, chronic unpredictable stress had no effects on dendritic morphology in the CA1, and reduced arborizations in the BLA^28^. Anxiety-like behaviour was only observed when contrasting patterns of dendritic remodeling were observed in the HPC and BLA^28^. This suggests that differential effects on synaptic remodeling between the HPC and BLA is an indicator of anxiety in stress models, although it is not yet known if it is a cause or consequence. Further, different neuronal cell types within the BLA may possess divergent responses to inflammation. For example, within the medial amygdala (MeA), stellate, but not bipolar (i.e., pyramidal) neurons, exhibited stress-induced plasticity, and structural changes in response to antidepressants^29^. Here, we sought to test the relationship among spine remodeling and evoked neural activity in HPC and BLA, and to correlate this with stress-coping behaviours in the DSS model of gut inflammation. We exposed mice to two different doses of DSS in the drinking water in order to produce varying degrees of intestinal inflammation. This produced differential effects on spine density and maturity, as well as excitability, in the HPC and BLA.

## Materials and methods

### Experimental animals and housing conditions

The studies were performed with 39 male C57BL6J mice (7–8 weeks of age, Jackson, Bar Harbour, ME, USA) housed in standard conditions (12:12 h light dark cycle, food and water ad libitum). Mice were habituated to the animal facility one week prior to any experimental manipulation. All experiments were carried out in accordance with the guidelines of the Canadian Council of Animal Care, were approved by the University of Lethbridge Animal Care Committee, and are in compliance with ARRIVE guidelines.

### Study design

This work included two experiments. In the first experiment, 24 mice were randomly allocated to three treatment groups (n = 8/group). They received either 2% DSS (MW 40 kD; Fisher Scientific, Ottawa, ON, Canada; CAT 9011-18-1) or 3% DSS added to the drinking water for 5 days, followed by exposure to regular drinking water for an additional 3 days; controls received drinking water throughout the experiment. Based on the results of the first experiment, we selected an intermediate dose (2.5% DSS) for the subsequent experiment. This was to ensure mice were sufficiently sick, while not losing an excessive amount (< 15%) of their body weight, as per veterinarian recommendations. Mice were exposed to 2.5% DSS (n = 9) for 5 days, or drinking water (n = 6). On day 8, animals were euthanized via i.p. injection of sodium pentobarbital (340 mg/kg; Merck & Co, Quebec, Canada).

### Forced swim task

The forced swim task (FST) was conducted on day 8, 60 min before mice were euthanized. Mice were placed in a clear glass cylinder (30 cm tall, 15 cm diameter) filled 14 cm deep with room-temperature water and video-recorded for 5 min. Mice were monitored continuously. Any animal unable to keep its head above water was immediately removed from the cylinder. After completing the task, mice were removed, dried with a paper towel, and placed in a cage with a heating pad before being returned to the home cage. Immobility time was defined by minimal movements to keep its head above water, recorded by an observer blind to the experimental treatment.

### Colitis assessment

After euthanasia, the colon was removed and macroscopic damage score calculated based on previously published criteria^20,30^, with minor modifications based on^31^. Body weight loss score was calculated as the percentage of body weight loss from baseline, multiplied by 0.2; colon shortening score was calculated as the percentage relative to negative controls multiplied by 0.05; colon inflammation score was calculated as the percentage of inflammation relative to colon length multiplied by 0.025. The absence (0) or presence (1) of erythema, fecal blood, and diarrhea, and degree of visceral adhesion (none = 0, minor adhesion = 1, major adhesions = 2) was recorded. The macroscopic damage score is the sum of body weight loss score, colon length score, colon inflammation score, colon thickness (mm), ulcer length (cm), and fecal blood, diarrhea, erythema, and adhesion score.

### Golgi labeling

In study 1, after euthanasia, animals were transcardially perfused with 0.9% saline. Brains were removed, impregnated with Golgi-Cox solution for 14 days, followed by immersion in 30% sucrose solution for 5 days^32^. Brain tissues were sectioned coronally via vibratome at 200 μm or 250 μm and mounted on a 2% gelatin-coated microscope slide. Slides were stored in a humidity chamber for a minimum of 12 h. Following this, slides were submerged in distilled water for 1 min, ammonium hydroxide for 30 min in the dark, and Kodak Fix for Film (McBain Camera, Lethbridge, AB, Canada) for 30 min in the dark. Slides were rinsed in distilled water and then dehydrated in alcohol (50% EtOH 1 min; 70% EtOH 1 min; 95% EtOH 5 min; 100% EtOH 5 min; 100% EtOH 5 min). Subsequently slides were cleared in a 1:1:1 Chloroform: HemoDe: 100% EtOH solution for 15 min, followed by 100% HemoDe for 15 min, and cover-slipped with Permount.

### Immunohistochemistry

In study 2, euthanized animals were transcardially perfused with 0.9% saline and 4% paraformaldehyde 60 min after completion of the FST. Brains were removed and fixed in 4% paraformaldehyde for 24 h, followed by cryoprotection in 30% sucrose for 48 h. Brains were embedded in Tissue Tek ® OCT medium (Fisher Scientific, Ottawa, ON, Canada) and stored at -80ºC until sectioning. Frozen brains were sectioned at 30 µm, and free-floating sections were blocked with 5% normal goat serum in antibody diluent. Every 12th section was incubated with the primary antibody rabbit anti-cFos (1:6400, Cell signaling 9F6, Whitby ON, Canada) for 24 h, the secondary antibody goat anti-rabbit (1:500, Invitrogen, Ottawa, ON, Canada) for 24 h, and counterstained with DAPI (1:2000, Sigma, St. Louis, MO, USA). Slides were mounted with permount, coverslipped, and stored at 4 °C until imaging.

### Imaging and analysis

In study 1, slides were examined and imaged with a microscope (Zeiss AxioPlan, with Retiga 2000R digital camera; Walpole, MA, USA) to assess the quality of staining and localize the hippocampus and basolateral amygdala. Based on this criteria, one to two sections from six brains per treatment group were selected for analysis. The sections were then imaged using laser scanning confocal microscopy (Leica DM5500 B, Concord, ON, Canada) with a 488 nm laser and 63X objective. Pyramidal neurons are the dominant neuronal phenotype in the CA1 region of the HPC. The BLA contains two principal cell types that can be differentiated based on morphological characteristics. Pyramidal BLA neurons bear a similarity to cortical pyramidal neurons, with clear apical and basal dendrites. Stellate neurons (similar to cortical spiny stellate cells) do not possess apical and basal designations, with dendrites radiating centrally from the soma. For analysis, three to four pyramidal neurons from the CA1 region of the hippocampus (Fig. 2a–d) and three to five pyramidal neurons from the BLA (Fig. 3a–d) were imaged from at least two sections per animal. One apical and one basal dendrite were analyzed per neuron; 20–30 dendrites were analyzed in total for each group. Additionally, one to four stellate neurons from the BLA were imaged from one to two sections per animal, with 12–19 dendrites analyzed for each group (control; 19 dendrites; 2% DSS, 15 dendrites; 3% DSS, 12 dendrites; Fig. 4a,b). Confocal image stacks were imported into FIJI software (version 1.53 g^33^). The measurement point function was used to identify approximately 20 μm of the distal end of a dendrite and the length of the segment was recorded. The grayscale image was inverted, and the image was sharpened. Dendritic spines were counted manually with the Cell Counter plugin. Spines were classified as head if a clear head could be identified, bifurcated if two branches of the spine could be identified, and immature if neither criteria were met (Figs. 2d, 3d, 4b). Analyses were completed by an observer blind as to the treatment status (2% DSS, 3% DSS, control) of the animal.

In study 2, brain sections were imaged at 40 × magnification using a Hamamatsu NanoZoomer 2.0 HT Scan System (Hamamatsu Photonics, Bridgewater, NJ, USA). For quantification of cFos expressing soma, both hemispheres from one coronal section containing the region of interest was selected by an experimenter blinded to the treatment status of the animal. The Ilastik 1.1.7 software cell density counting program and Image J 1.4.3.67 software^34,35^ was used for quantifying the total number of cells expressing immunofluorescence for cFos, and the area of the region of interest. Analysis from three animals was excluded due to non-specific staining as a result of poor perfusions.

### Data presentation and statistical analysis

Data are presented as mean ± SEM for normally distributed data, or median and 95% confidence intervals for non-normally distributed data. Statistical significance was set at p < 0.05. Outliers were removed using ROUT analysis (Q = 1%) and normality was assessed via Shapiro–Wilk test. Differences between means/medians were compared using a student’s t-test, One-Way ANOVA (Dunnett’s post-test), Two-way ANOVA (with Tukey’s post-test), or Kruskal Wallis (Dunn’s post-test). Linear regression was used to assess relationships between macroscopic damage score, immobility in the FST, and cFos expression.

## Results

### Colonic damage and body weight loss depend on DSS dose

**Figure 1:**
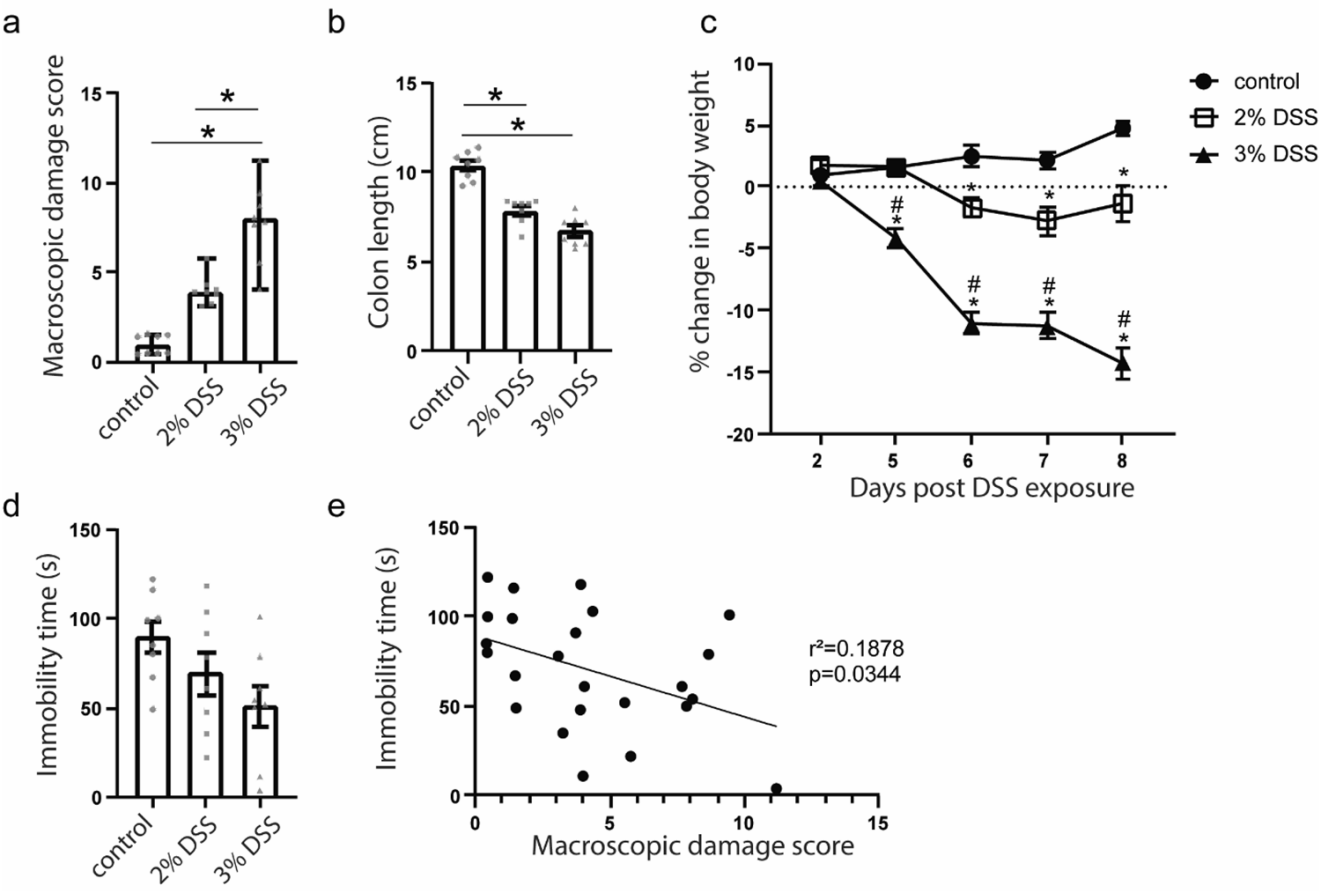
Exposure to DSS promotes colon shortening, weight loss, and macroscopic damage in the colon, which is negatively correlated with reduced immobility time in the forced swim task. (**a**) Macroscopic damage score; Kruskal Wallis, Dunn’s post-test *p < 0.05 relative to control. Data shown are median ± 95% CI. (**b**) Colon length; One Way ANOVA, Dunnett’s post-test, *p < 0.05 relative to control. Data shown are mean ± SEM. (**c**) Change in body weight over course of DSS exposure; two-way ANOVA, Tukey’s post-test; *p < 0.05 versus control, ^#^p < 0.05 versus 2%DSS treatment. Data shown are mean ± SEM. (**d**) Total immobility time in the forced swim task; One-way ANOVA, p = 0.0627. Data shown are mean ± SEM. (**e**) Immobility time as a function of macroscopic damage score; linear regression. N = 8 per group.

The addition of DSS to drinking water evokes several pathophysiological features in the colon, which are often aggregated into a weighted sum called the macroscopic damage score. Disease severity depended on the concentration of DSS added to the drinking water. The macroscopic damage score of mice exposed to 2% DSS (Median = 3.91 (3.370–4.283); *p* = 0.0324) and 3% DSS (Median = 7.965 (6.085–9.258); *p* < 0.0001) were significantly higher than for controls (Median = 0.91 (0.4425–1.465); (Main effects Kruskal Wallis H = 19.29, *p* < 0.0001; Fig. 1a), but were not significantly different from each other (*p* = 0.1430). Colon length was significantly shorter in mice exposed to 2% and 3% DSS relative to controls (one way ANOVA, F_(2,21)_ = 47.12 *p* < 0.0001), and relative to each other (*p* = 0.0176; Fig. 1b). Both 2% and 3% DSS exposure was associated with weight loss throughout the course of the experiment (Two-way ANOVA, main effect of time F_(2.015,42.32)_ = 36.77 p < 0.0001; main effect of treatment F_(2,21)_ = 74.38 p < 0.0001). Greatest body weight reductions were observed in the 3% DSS treatment group (Fig. 1c). Relative to baseline, controls gained 4.76 ± 0.638% of their body weight on day 8, while animals in the 2% DSS and 3% DSS treatment lost 1.33 ± 1.465% and 14.26 ± 1.182% of their body weight, respectively (Fig. 1c). These data indicate a dose-dependent effect of DSS on colon structure and gut function.

### Severity of macroscopic damage of the colon is negatively correlated with immobility time in the FST

We next investigated the effects of DSS on stress-coping behaviours in the FST. Exposure to DSS was associated with a non-significant trend towards reduced immobility time in the FST (One-way ANOVA, F_(2,21)_ = 3.170, p = 0.0627; Fig. 1d). When data was pooled across all treatment groups, we observed that the severity of macroscopic damage was negatively correlated with immobility time in the FST (F_(1,22)_ = 5.087, p = 0.0344; linear regression, r^2^ = 0.1878 Fig. 1e).

### 3% DSS administration increases proportion of immature spines in CA1 HPC

**Figure 2:**
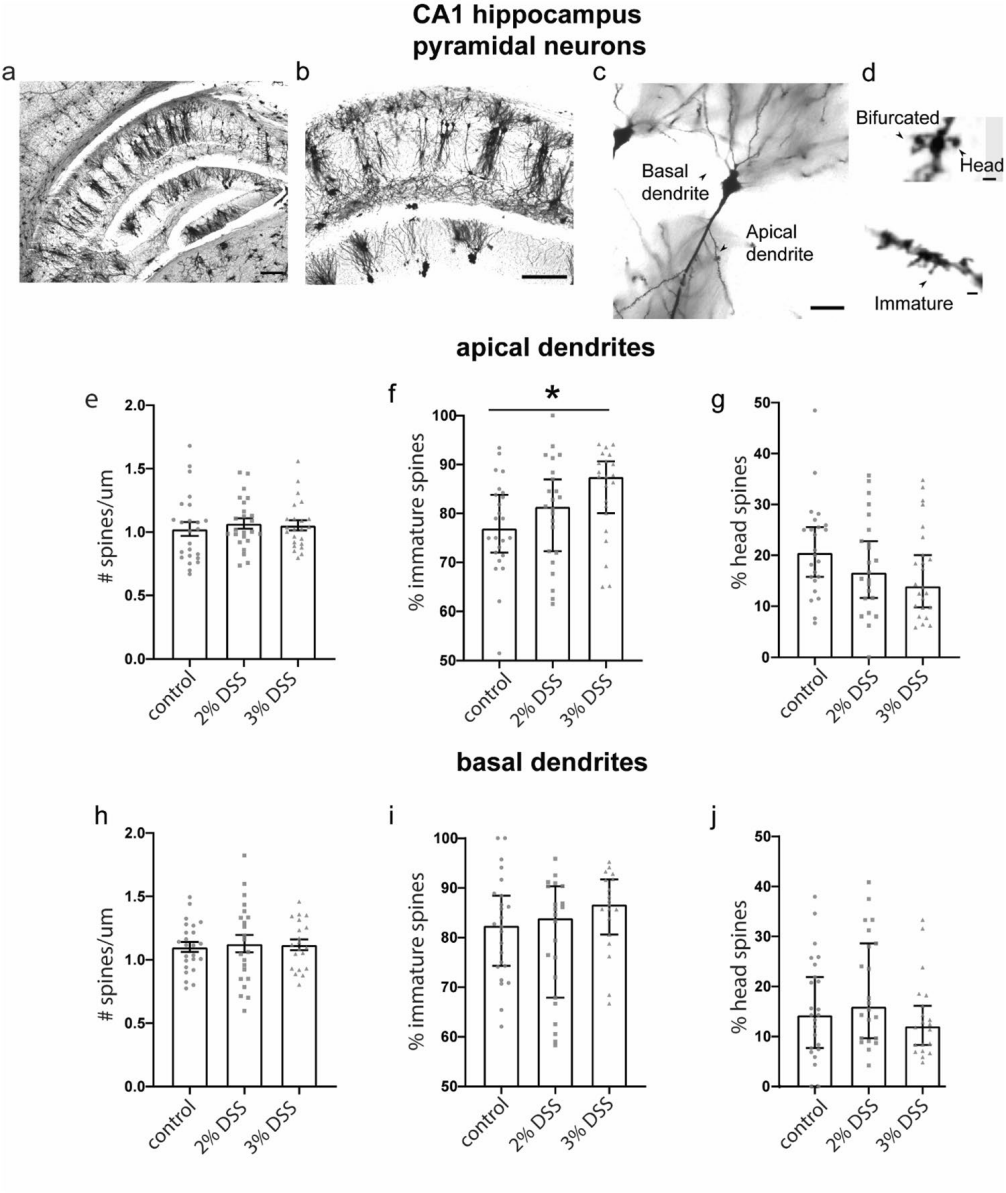
Spine density and composition on basal and apical dendrites of CA1 neurons in the hippocampus. (**a**) Photomicrograph of Golgi labeled cells of CA1 region in the mouse hippocampus (scale bar: 200 µm). (**b**) Larger view of panel a (scale bar: 200 μm). (**c**) Representative image of CA1 neuron (scale bar: 25 µm). (**d**) Representative examples of spine morphologies in CA1 (scale bar: 1 µm). (**e**) Density of spines on apical dendrites in CA1; data shown are mean ± SEM. Proportion of immature spines (**f**) and head spines (**g**) on apical dendrites in the CA1; data shown are median ± 95% CI. (**h**) Density of spines on basal dendrites in CA1; data shown are mean ± SEM. Proportion of immature spines (**i**) and head spines (**j**) on basal dendrites in the CA1; data shown are median ± 95% CI. Density data analyzed by One-way ANOVA. Composition data analyzed by Kruskal Wallis with Dunn’s post-test to negative control; *p < 0.05. n = 20–24 dendrites, from 6 neurons, from 6 animals per group.

We then investigated whether the dose of DSS was associated with changes in spine density or morphology. Previous research has indicated that basal and apical dendrites have different sensitivities to treatments^36–38^, possibly due to their partially dissociated mechanisms of long term potentiation^39^. Therefore, spine density and morphology of apical and basal dendritic branches were analyzed separately. Exposure to DSS did not impact apical (F_(2,63)_ = 0.07122, p = 0.9313) or basal (One-way ANOVA, F_(2,68)_ = 0.2376, p = 0.7892) spine density in the CA1 (Fig. 2e,h). However, the proportion of immature spines on apical dendrites increased with DSS (non-normally distributed data; Kruskal Wallis, H = 6.164, p = 0.0459) which was significantly greater among mice exposed to 3% DSS relative to controls (p = 0.0277), but not 2% DSS (p = 0.7291 Fig. 2f). Consistently, the proportion of head spines tended to reduce with increased DSS concentration (median proportion of head spines for control = 20.51%, 2% DSS = 16.65%, 3% DSS = 13.98%) on apical dendrites in the CA1 (Fig. 2g), though this did not reach significance (Kruskal Wallis, H = 3.152, p = 0.2608). No differences in the proportion of bifurcated spines were observed between groups (Kruskal Wallis, H = 2.269, p = 0.3216). The shift towards an increased proportion of immature spines among DSS-treated animals was not observed in spines on basal dendrites (immature, Kruskal Wallis H = 2.848, p = 0.2408, Fig. 2i; head, H = 3.644, p = 0.1617, Fig. 2j; bifurcated, H = 2.812, p = 0.245, data not shown).

### Exposure to 2% DSS reduced spine density on dendrites of stellate but not pyramidal neurons in the BLA

**Figure 3:**
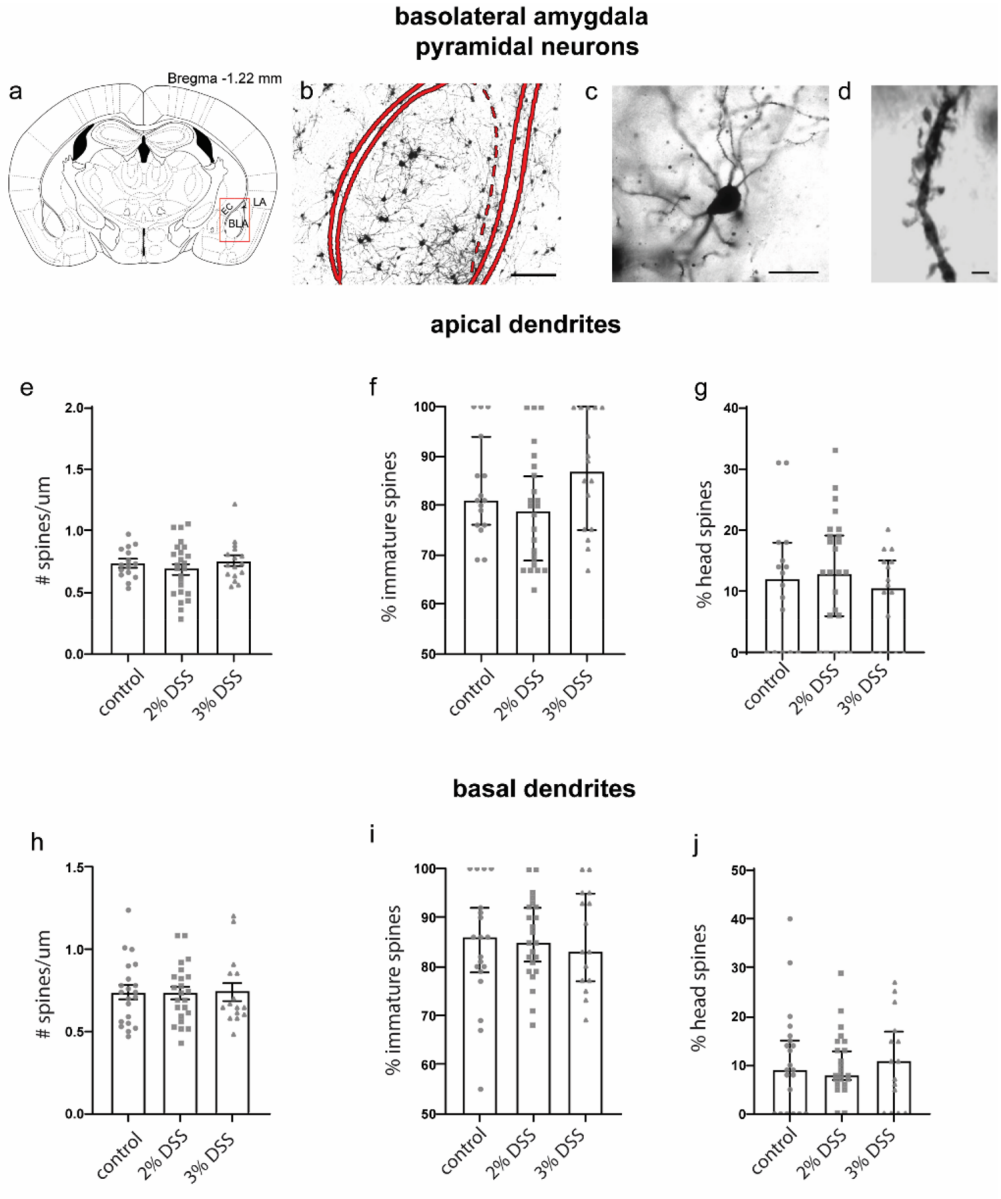
Spine density and composition on basal and apical dendrites of pyramidal cells in the basolateral amygdala. (**a**) Region of interest illustrated on a standard mouse brain atlas, (**b**) Photomicrograph of Golgi labeled cells in basolateral amygdala (BLA) region in a mouse brain (scale bar: 200 µm). (**c**) Representative image of a BLA pyramidal neuron (scale bar: 25 µm) (**d**) Representative image of dendrite selected for spine counting (scale bar: 2 µm). (**e**) Density of spines on basal dendrites in BLA; data shown are mean ± SEM. Proportion of immature spines (**f**) and head spines (**g**) on basal dendrites in the BLA; data shown are median ± 95% CI. (**h**) Density of spines on apical dendrites in BLA, data shown are mean ± SEM. Proportion of immature spines (**i**) and head spines (**j**) on apical dendrites in the BLA; data shown are median ± 95% CI. Density data analyzed by One-way ANOVA with Dunnett’s post-test to negative control, data shown as mean ± SEM, *p < 0.05. Composition data analyzed by Kruskal–Wallis. n = 15–24 dendrites, from 12 to 19 neurons, from 5–6 animals per group.

**Figure 4:**
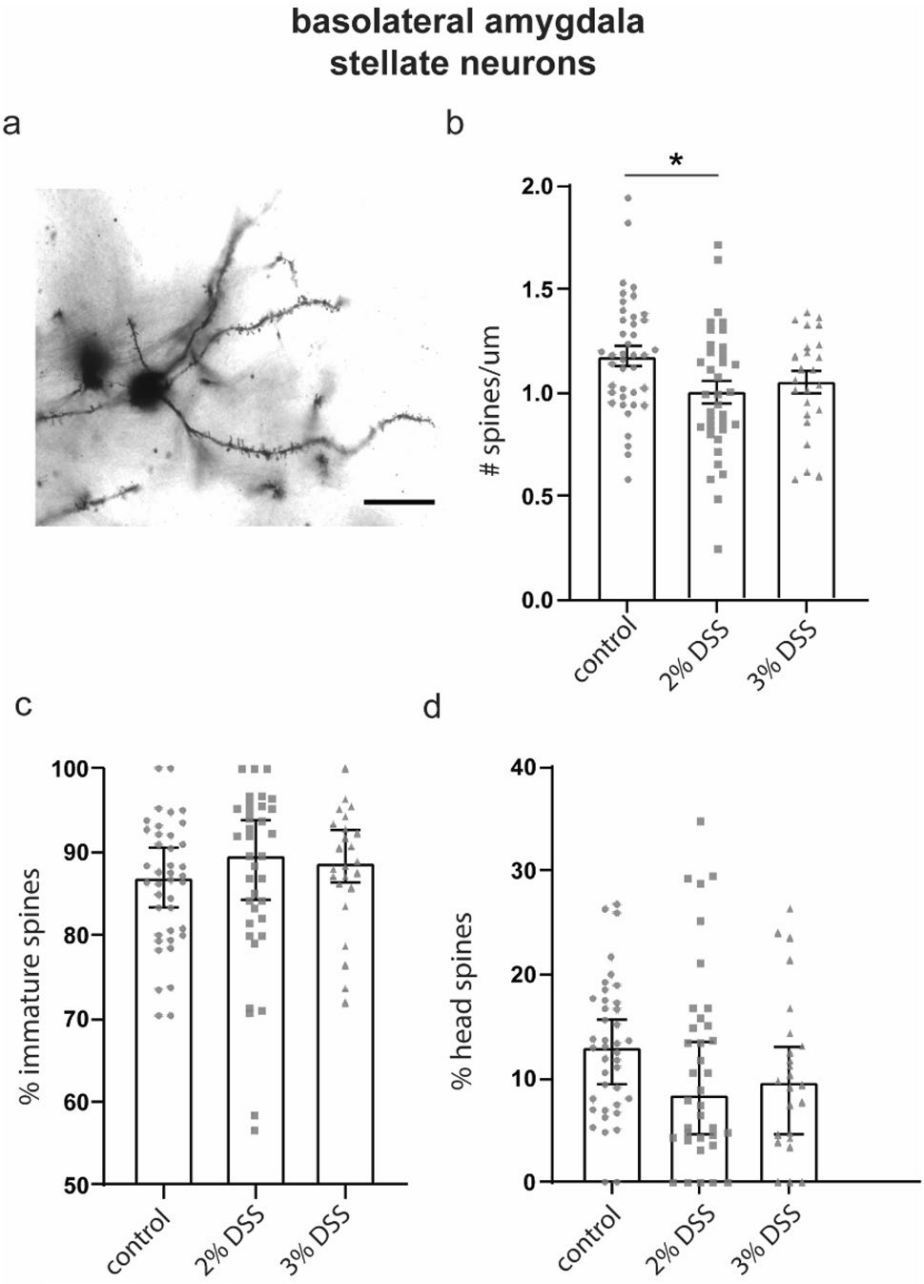
Spine density and composition of stellate neurons in the basolateral amygdala. (**a**) Representative image of a stellate neuron; scale bar 25um. (**b**) Density of spines on dendrites in BLA; data shown are mean ± SEM. Proportion of immature spines (**c**) and head spines (**d**) on dendrites in the BLA; data shown are median ± 95% CI. Density data analyzed by One-way ANOVA with Dunnett’s post-test to negative control, data shown as mean ± SEM, *p < 0.05. Composition data analyzed by Kruskal–Wallis. n = 24–40 dendrites, from 12 to 19 neurons, from 6 animals per group.

We next examined spine density and morphology on pyramidal and stellate neurons in the BLA of mice exposed to DSS (Figs. 3, 4). Spine density on pyramidal basal dendrites was not affected by treatment (Kruskal Wallis H = 0.6806, p = 0.7115; Fig. 3e), neither was the proportion of immature (One way ANOVA F_(2,54)_ = 0.1006, p = 0.9044, Fig. 3f), head (Kruskal Wallis H = 0.001566 p = 0.9992, Fig. 3g) or bifurcated (Kruskal Wallis H = 2.767, p = 0.2507, data not shown) spines. Similarly, treatment did not affect spine density (One way ANOVA; F_(2,53)_ = 0.6706, p = 0.5157) or spine morphology on apical dendrites of pyramidal cells in the BLA (immature, Kruskal Wallis H = 4.456 p = 0.1077, Fig. 3i; head, Kruskal Wallis H = 2.144 p = 0.3424, Fig. 3j; bifurcated, H = 2.505, p = 0.2858, data not shown). However, we observed a significant reduction of spine density on stellate neurons among 2% DSS (p = 0.0149), and we observed a trend towards reduced spine density among 3% (p = 0.1508) DSS-treated animals relative to controls (One-way ANOVA, F_(2,98)_ = 3.933, p = 0.0227, Fig. 4a–d), although this did not reach statistical significance. Treatment had no effect on the proportion of bifurcated spines (Kruskal Wallis H = 2.154, p = 0.3406; data not shown).

### cFos expression in the CA1 is reduced among animals treated with DSS, negatively correlated with macroscopic damage, and positively correlated with immobility time

**Figure 5:**
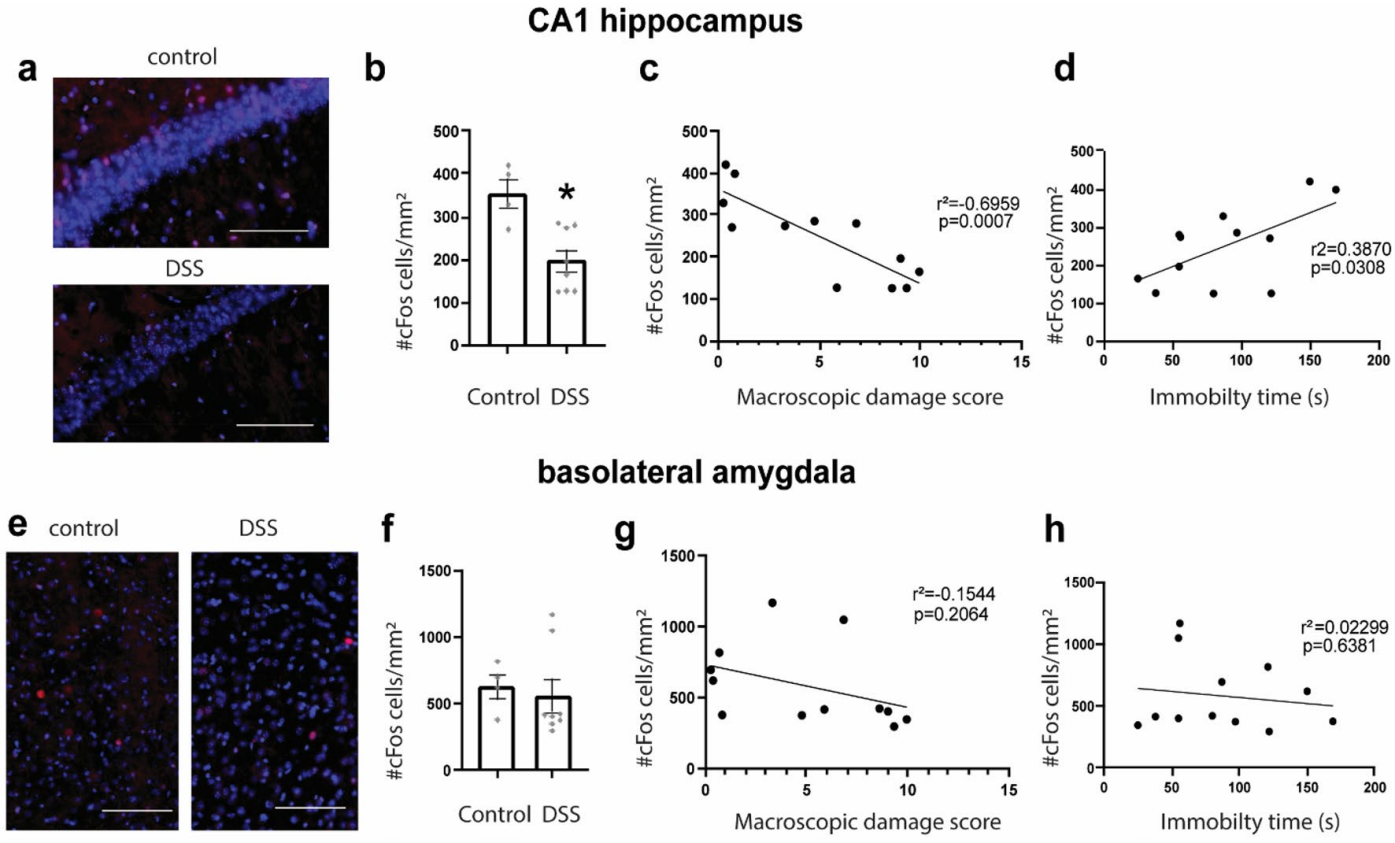
cFos expression in the CA1 hippocampus and the basolateral amygdala. (**a**) Representative image of cFos expression of control and DSS-treated animals in the CA1 of the hippocampus. DAPI in blue, cFos in red. Scale bar 100 µm. (**b**) Density of cFos^+^ cells in the CA1 of the hippocampus; student’s t-test; *p < 0.05. (**c**) Linear regression between cFos density in the CA1 and macroscopic damage score. (**d**) Linear regression between cFos expression in the CA1 and immobility time. (**e**) Representative image of cFos expression of control and DSS-treated animals in the BLA. DAPI in blue, cFos in red. Scale bar 100 µm. (**f**) Density of cFos^+^ cells in the BLA. (**g**) Linear regression between cFos density in the BLA and macroscopic damage score. (**h**) Linear regression between cFos density in the BLA and immobility time Data are mean ± SEM Control (n = 4) and 2.5% DSS (n = 8).

In a second cohort of mice (n = 15), we sought to assess how exposure to DSS affected the proportion of neurons activated in CA1 HPC and BLA during the FST. Macroscopic damage, body weight, colon length, and other behavioural tasks for this study are reported elsewhere^30^. We used cFos expression 60 min after the forced swim task as an indicator of neural activation (Fig. 5). Exposure to 2.5% DSS significantly reduced the expression of cFos^+^ cells per mm^2^ in CA1 (Student’s t test = 3.609; df = 10; p = 0.0048 Fig. 5b), but not in BLA (Student’s t test = 0.3646, df-10; p = 0.7230, Fig. 5f). Further analysis revealed a significant negative correlation between the density of cFos expression in the CA1 and macroscopic damage score for each mouse (Linear regression F_(1,10)_ = 22.89, p = 0.0007, r^2^ = 0.6959 Fig. 5c) among control and DSS-treated mice; this correlation was not observed in the BLA (Fig. 5g). Finally, there was a positive correlation between CA1 cFos expression and immobility time during the FST (Linear regression F_(1,10)_ = 6.313, p = 0.0308, r^2^ = 0.3870 Fig. 5d), but not the BLA (Linear regression F_(1,10)_ = 0.2523, p = 0.6381, Fig. 5h).

## Discussion

The data presented here suggest that there are region-specific and cell-specific effects of gut inflammation on spine remodeling and neural activity in the BLA and HPC. Results indicate that synaptic remodeling in these structures is influenced by the severity of gut inflammation and dendrite location (apical vs basal). Additionally, we observed cell-specific changes in gut inflammation-induced spine density in the BLA. Furthermore, activity in the HPC, but not BLA, was reduced in mice with colitis, and the reduction in activity correlated with changes in behavior, suggesting increased activation of active threat coping strategies.

Animal models of gut inflammation are associated with a suite of behavioural changes including increased anxiety-like behaviours, depressive-like behaviours, and modified stress responses^4,16,17,20,40–43^. Recent evidence suggests the FST, which was traditionally employed as a measurement of behavioural despair and helplessness, can be used to measure stress-coping strategies^44^. In the present study, we observed that colitis was associated with a correlative trend towards reduced immobility time in the FST. In other words, colitic mice showed more active swimming (and less passive floating) than controls. This is consistent with previous studies reporting that acute colitis increased active coping strategies in stressful situations^16,30^. Note, in the present study that we tested at peak of disease^13^. Other work assessing behaviour at a later time point, during the post-inflammatory phase of DSS colitis, suggest a shift toward passive stress-coping among male and female mice^45^ and rats^46^. Sex effects might also be an important consideration when testing for stress-coping strategies at the peak of disease. This is an important future consideration, as female mice are reported to exhibit increased passive stress coping during active gut inflammation^42^, as measured by immobility time in the FST. It should be mentioned that DSS-induced colitis drives weight loss in mice, and the relative contributions of gut inflammation and weight loss in the FST is not known. Combined, these data suggest that inflammation-driven changes in stress coping strategy depend on sex, severity of disease, and inflammatory stage (active versus recovery). Endocrine and other signaling molecules likely play a role. Studies of individual differences of stress-coping indicate that region-specific glucocorticoid and neuropeptide expression influence active versus passive strategies^47,48^.

Behavioural responses to stressful situations are mediated in part by the hypothalamic-pituitary axis (HPA), which is activated by both DSS colitis^43^ and the FST^44^. Both the HPC and BLA modulate HPA activity through their inputs onto the periventricular nucleus (PVN) of the hypothalamus. Anatomical^49–51^ studies reveal that HPC inhibits output of the PVN via excitatory inputs on GABAergic interneurons. Conversely, amygdala inputs are disinhibitory on PVN output, by reducing PVN GABAergic tone. Thus, HPC and the amygdala can exert opposing regulation of the HPA axis. The present data are consistent with this opposing regulation. The reduced cFos expression in CA1 of DSS-treated animals suggests the possibility of reduced HPC inhibition of PVN, which would increase HPA activation and promote active stress coping. Indeed, the reduction of CA1 cFos correlated with reduced immobility time on the FST. The colitis mediated reduction of hippocampal cFos is consistent with previous literature; both basal levels of cFos, and stress-evoked expression of cFos, are reduced in the CA1 of mice with gut inflammation relative to controls^52–54^. We found no effect of colitis on BLA activation. We were unable to identify prior literature regarding the effects of gut inflammation on BLA activity. In sum, our data support the possibility that DSS-induced colitis reduces HPC activity, which disinhibits PVN, to increase the HPA and promote active responses (swimming) to stress. Additional studies are required to test this hypothesis.

The opposing effects of colitis on activity between HPC and BLA were recapitulated in spine morphology. The relative proportion of immature spines in CA1 increased with severity of colitis. Mature spines are more likely to generate post-synaptic currents than immature ones^55^. The reduction in mature spines in DSS-treated mice, therefore, suggests that CA1 neurons have less synaptic drive, which could account for their reduced activity, as indicated by lower density of cFos positive cells. We did not find a dose-dependent change of spine maturity or neural activity in BLA. Such dissociation of effects between CA1 and BLA have been observed in some models of chronic stress. In particular, stressors that increase anxiety-like behaviours reduce spine density in the CA1, but increase spine density in BLA^24,56–60^. It is worth noting that many of the stressors are chronic, as opposed to the acute model of inflammation used in the present study. It is possible that some portion of the immature spines in CA1 that fail to mature will disappear at later time points, which would result in hypotrophy, consistent with chronic stress data.

Our finding that gut inflammation promotes spine remodeling in apical but not basal CA1 dendrites suggests a differential sensitivity among these domains. Limited evidence supports this assessment. For example, certain antidepressants^61^ and brain-derived neurotrophic factor (BDNF)^62^ have been shown to increase spine density in apical, but not basal dendrites of CA1 pyramidal neurons. Cranial irradiation studies revealed that spine morphology on apical and basal dendrites of CA1 neurons was significantly and differentially modulated, with spines on apical dendrites shifting towards a greater proportion of mature spines, and basal spines shifting towards a higher proportion of immature phenotype. Further, apical spine density on CA1 neurons was resilient to cranial irradiation-induced changes, while basal spine density was not^38^. Conversely, repeated exposure to the cholinesterase inhibitor paroxon in neonatal rats^36^, and normal aging processes in mice^36^ selectively reduced spine density in basal, but not apical dendrites of CA1. These data illustrate the importance of distinguishing between apical and basal domains when assessing spine remodeling in the HPC.

The differential effect of DSS on basal and apical spines begs the question: what is the mechanism and functional significance of this discrepancy? The fact that all pyramidal CA1 dendrites are separated into distinct apical and basal domains implies functional specialization, although the nature and degree of heterogeneity are unclear^63^. Apical and basal dendrites possess different morphologies, afferent inputs, and ion channel distribution^64^. Recent research reveals that long-term potentiation in the basal and apical domains have at least some distinct mechanisms^39,65–69^. This suggests that some molecular mechanisms of synaptic plasticity differ among apical and basal domains, which may also be differentially susceptible to effects of gut inflammation.

The observed cell-specific effects of gut inflammation between stellate and pyramidal BLA neurons similarly imply differential susceptibility to spine remodeling. Evidence suggests stellate neurons possess unique electrophysiological properties relative to pyramidal cells in the BLA. For example, stellate neurons possess a significantly larger cell soma, have a lower input resistance, shorter time of constant membrane charging, shorter duration of action potential, and a bursting, rather than sustained, firing pattern, relative to pyramidal BLA cells^70^. More recently, it was reported that pyramidal BLA neurons, but not stellate neurons, were responsive to antidepressant-induced spine remodeling^29^. Although it is tempting to speculate that the mechanisms that render stellate neurons more responsive to gut inflammation and less responsive to antidepressants are linked, this remains unknown. Mice exposed to 2% DSS, but not 3% DSS, possessed reduced spine density on stellate neurons in the BLA relative to controls. The absence of statistical significance between 3% DSS and control groups may be a consequence of the unequal sample sizes among treatment groups. Specifically, fewer dendrites were available for analysis in the 3% DSS-treated mice, relative to 2% DSS and control groups (see methods), due to the inherent limitations of golgi staining and confocal resolution. Another limitation of the present study is that we did not consider exposure to DSS might influence dendritic branching or complexity, which could reveal further insight into how gut inflammation promotes structural changes in the brain.

Animal models of gut inflammation are associated with increased expression of pro-inflammatory mediators (e.g. TNF), and reduced expression of BDNF^4,14,16,17,21,45^ in the HPC. Literature suggests altered levels of these factors in the CA1 likely affect spine density and/or morphology. For example, TNF was associated with reduced cortical spine density^71^, was found to mediate spine remodeling in a model of peripherally-induced neuroinflammation^22^, and has been shown to enhance loss of basal dendritic spines on CA1 neurons in a model of sleep deprivation^72^. Evidence from in vitro studies suggests that soma-derived BDNF increases spine density^73^, and some BDNF splice variants were reported to differentially modify apical vs basal dendrite spine morphology in cultured hippocampal neurons^74^. This is one possible mechanism that could play a role for the selective spine remodeling on apical dendrites; indeed, elevated BDNF increased spine density on apical, but not basal dendrites in CA1 neurons^62^.

The HPC and BLA communicate with many other brain regions. They are primary components of the so-called limbic system, which is fundamentally important for a variety of behaviours^5^. They both connect with the medial prefrontal cortex, including the anterior cingulate cortex. This tripartite network is proposed to be important for the contextual detection and behavioural responses to stimuli associated with negative (fearful or aversive) and positive (satisfaction) affective states^5^. The acute gut inflammation remodeling of HPC and BLA neurons reported here supports proposals^4^ that inflammation and pain in gastrointestinal disorders combine to functionally re-organize circuits in this network to promote anxiety and stress-coping behavioural phenotypes, partly by increasing the reactivity of the HPA.

## Data Availability

The datasets used and/or analyzed during the current study available from the corresponding author upon reasonable request.
